# Endocrine Risk Factors of Endometrial Cancer: Polycystic Ovary Syndrome, Oral Contraceptives, Infertility, Tamoxifen

**DOI:** 10.3390/cancers12071766

**Published:** 2020-07-02

**Authors:** Atanas Ignatov, Olaf Ortmann

**Affiliations:** Department of Gynecology and Obstetrics, University Medical Center Regensburg, Landshuter Str. 65, 93053 Regensburg, Germany; oortmann@caritasstjosef.de

**Keywords:** endometrial cancer, PCOS, tamoxifen, infertility, oral contraceptives

## Abstract

Endometrial cancer is the most common gynecologic cancer and is predominantly endocrine-related. The role of unopposed estrogen in the development of endometrial cancer has been investigated in numerous studies. Different reproductive factors such as younger age at menarche, late age at menopause, infertility, nulliparity, age of birth of the first child, and long-term use of unopposed estrogens during hormone replacement therapy have been associated with an increased risk of endometrial cancer. In contrast, there is a growing body of evidence for a protective role of oral contraceptives. Most of the published data on the association between infertility and polycystic ovary syndrome are inconclusive, whereas the effect of tamoxifen on the risk of endometrial cancer has been well established. With this review, we aim to summarize the evidence on the association between infertility, polycystic ovary syndrome, oral contraceptives, and tamoxifen and the development of endometrial cancer.

## 1. Introduction

Endometrial cancer is the most common gynecologic cancer [1]. There are two different types of endometrial cancer: estrogen-related type I, comprising about 80% of the diagnosed endometrial cancers and a smaller group of non-estrogen-related type II cancer. One of the most important characteristics of type I endometrial cancer is its estrogen dependence. Estrogen stimulates the proliferation and growth of type I endometrial cancer cells, whereas progesterone and synthetic derivatives have the opposite effect. Thus, several pathophysiological conditions, associated with increased estrogen levels, might increase the risk of type I endometrial cancer. In this regard, medication influencing the estrogen/progesterone ratio might also influence the incidence of endometrial cancer. Various reproductive factors such as younger age at menarche and late age at menopause, infertility, nulliparity, age at first delivery, and long-term use of unopposed estrogens during hormone replacement therapy has been associated with a significantly increased risk of developing endometrial cancer [2]. On the other hand, increased parity and the use of combined oral contraceptives (OC) as well as the use of progestins for contraceptive purposes have been shown to reduce the risk of endometrial cancer.

The aim of this review is to investigate the relationship between infertility, polycystic ovary syndrome (PCOS), OCs, and tamoxifen (TAM) and the risk of endometrial cancer.

## 2. Infertility and Risk of Endometrial Cancer

Various previous studies suggested a correlation between nulliparity and endometrial cancer [2]. However, whether this correlation is due to infertility itself or rather to other factors associated with infertility remains unclear. In addition, the extent of this correlation is controversial due to the heterogeneity of these studies. Furthermore, the influence of different confounders associated with infertility has been poorly investigated. More recently, several large observational studies assessing infertility and its association with endometrial cancer have been published.

For example, a pooled analysis of 2 cohort studies and 12 case–control studies with 8153 cases and 11,713 controls investigated the association between infertility and incident endometrial cancer [3]. In all of these studies except for one, infertility was documented by self-reporting. This pooled analysis demonstrated that nulliparous women have a significantly elevated risk of endometrial cancer compared with parous women (odds ratio [OR] 1.76; 95% confidence interval [CI] 1.65–2.00) (Table 1).

This risk was similar after adjustment for infertility (OR 1.82; 95% CI 1.59–1.94). Infertility itself was also associated with an increased risk of endometrial cancer risk, with OR of 1.22 (95% CI 1.13–1.33) and 1.20 (95% CI 1.11–1.30) after adjustment for parity and number of deliveries, respectively. Furthermore, the influence of several other putative risk factors of endometrial cancer was investigated. For example, a significantly elevated risk was found for anovulation/PCOS among nulliparous women, whereas an association between endometriosis and endometrial cancer risk was not statistically significant. These data demonstrate that parity and infertility independently contribute to endometrial cancer risk [3]. In addition, the available data suggest that nulliparity is a stronger predictor than infertility.

The strengths of the study by Yang et al. are the large sample size and the fact that in most cases the confounding factors were adequately considered. Nevertheless, several limitations should be mentioned. The self-reported infertility raises the question of potential bias. Furthermore, the infertility definition was not consistent across the included studies, and there was a significant difference in the prevalence of infertility among cases and controls. The influence of fertility treatment was only investigated in a small number of patients. Since infertility treatment has been reported to potentially influence endometrial cancer risk [4], its consideration as a confounder is important and should be taken into account. For example, the risk of endometrial cancer and the use of assisted reproductive techniques has been investigated in a very large cohort study [5]. Compared with the general population, the risk of endometrial cancer was increased in women receiving clomiphene citrate (HR: 2.91, 95% CI 1.87–4.53) but not in those undergoing in vitro fertilization (HR: 1.62, 95% CI 0.70–3.85). This study is limited by the lack of control for confounding factors. Nevertheless, the use of infertile women as controls will help us to adjust for preexisting endometrial cancer risk factors that may exist in infertile women. In addition to the infertility treatment, the unopposed estrogen effect is another important mechanism increasing the risk of endometrial cancer.

Recently, further observational studies have looked into the issue of infertility and endometrial cancer. For example, in a nationwide population-based cohort study, Lundberg et al. investigated the risk of breast and gynecological cancers among women with a diagnosis of infertility [6]. They found that among 2,882,847 Swedish women, infertility was associated with an increased risk of endometrial cancer. Specifically, infertility was associated with a higher incidence rate of ovarian (adjusted hazard ratio [aHR] 1.53; 95% CI 1.38–1.71) and endometrial cancer (aHR 1.25; 95% CI 1.11–1.40), but not of breast cancer (aHR 0.96; 95% CI 0.92–1.01). Similar to the aforementioned study by Yang et al., the incidence of endometrial cancer was elevated among women with ovulatory disturbances but not among women with endometriosis. Infertility was diagnosed in 4.1% of women included in this cohort, and the higher incidence of endometrial cancer was restricted to women with both infertility and ovulatory disturbances (aHR 2.90; 95% CI 2.05–4.08). The association between infertility and ovulatory disturbances and endometrial cancer appeared to be stronger in nulliparous women or in younger women (<50 years). These data suggest that the elevated endometrial cancer risk is more likely due to ovulatory dysfunction than to infertility per se.

One of the most important drawbacks of the study by Lundberg et al. is the missing data regarding infertility, body mass index (BMI), and the use of OCs. BMI and OCs might significantly influence the risk of endometrial cancer among women with infertility and should therefore be considered relevant confounders.

In another recent study, the risk of developing gynecologic cancers was analyzed in infertile US women using claims data [7]. In this large retrospective study 64,345 infertile women were compared to 3,128,345 non-infertile women seeking routine gynecologic care. The result of this study was that infertile women had a significantly elevated risk of developing gynecologic cancers, in particular uterine cancer, compared to non-infertile women. Patients in the infertile group were older and were more likely to be nulliparous and smokers compared to non-infertile women. However, this potential selection bias was accounted for by using a multivariate analysis. Doing so, the adjusted risk for endometrial cancer was significantly higher among infertile women compared to fertile controls (aHR 1.78; 95% CI 1.39–2.28). The adjustment included factors such as age at index date, nulliparity, race, smoking, obesity, level of education, and number of visits per year. Notably, 36.3% of infertile women had a diagnosis of PCOS or endometriosis, whereas in the fertile control group only 4.0% were diagnosed with PCOS or endometriosis. In addition, the authors performed a subset analysis excluding patients with PCOS and endometriosis from both groups. Notably, the overall risk of gynecologic cancers remained higher for infertile women, but not the risk of endometrial cancer (aHR 0.99; 95% CI 0.64–1.55). These data clearly demonstrate that the influence of various reproductive confounders has to be considered if the association between infertility and endometrial cancer is investigated. Besides the imbalanced distribution of the risk factors in both groups, further important limitations of the study were the low absolute rate of cancer, the short follow-up, and the known limitations of studies based on data derived from insurance databases.

In summary, when taking into account the available data and the limitations of most studies, a reliable conclusion regarding the association between infertility and the risk of endometrial cancer should be made with caution. The most important challenges in analyzing such data are the low incidence of malignancies in reproductive-age women, a short follow-up in the included studies, the heterogeneity of the study populations, and the influence of additional reproductive factors such as PCOS, endometriosis, or infertility treatment, which were accounted for. In particular, the effect of infertility treatments on the risk of endometrial cancer is the topic of an ongoing debate. The available data provide somewhat conflicting results [4]. For example, in a meta-analysis of cohort and case control studies, Skalkidou et al. summarized the incidence of endometrial cancer among 1.9 million women undergoing various infertility treatments. Fifteen eligible studies, using a general population as the control group, found an increased risk after exposure to any ovary-stimulating drug (relative risk [RR] 1.75; 95% CI 1.18–2.61). Four studies found an increased risk of endometrial cancer in subfertile women who required clomiphene citrate compared to a general population control group (RR 1.87; 95% CI 1.00–3.48). Exposure to clomiphene citrate as an ovary-stimulating drug in subfertile women is associated with increased risk of endometrial cancer, especially at doses greater than 2000 mg and high (more than seven) number of cycles. This may largely be due to underlying risk factors in women who need treatment with clomiphene citrate, such as polycystic ovary syndrome, rather than exposure to the drug itself. Lastly, exposure to gonadotropins was also associated with an increased risk of endometrial cancer (RR 1.55; 95% CI 1.03–2.34).

In summary, a thorough analysis of the data especially regarding different control groups strongly suggests that the key factor triggering endometrial cancer risk might be infertility and the factors leading to and associated with infertility, rather than infertility treatment itself.

## 3. PCOS and Risk of Endometrial Cancer

The hormonal imbalance caused by anovulation in patients with PCOS is associated with unopposed estrogen action [2]. Estrogen-driven proliferation and differentiation might then lead to the development of endometrial hyperplasia and ultimately endometrial cancer. Moderate-quality data showed that women with PCOS have a 2.7-fold increased life-time risk for developing endometrial cancer.

In the last decade, the association between PCOS and endometrial cancer has been investigated in numerous studies. In a meta-analysis of eight eligible studies published between 1968 and 2008, an almost three-fold increased risk of endometrial cancer for women with PCOS was documented [7]. In another meta-analysis including 919 women with PCOS and 72,054 non-PCOS controls, Barry et al. also found an increased risk of endometrial cancer among women with PCOS [8]. The estimated OR was 2.79 (95% CI 1.31–5.95). The authors suggested that the increased risk for endometrial cancer might be influenced by the increased prevalence of obesity in women with PCOS. Supporting this, two studies investigating BMI as a confounder did not observe any association between PCOS and endometrial cancer. Specifically, in a study with women ≤50 years, 156 cases and 398 controls were compared [9]. A fourfold increased risk of endometrial cancer in women with PCOS compared to women without PCOS (OR 4.0; 95% CI 1.7–9.3) was observed. Notably, this association was no longer significant after adjustment for BMI (OR 2.2; 95% CI 0.9–5.7). In another case–control study, the role of different reproductive factors and their association with endometrial cancer were investigated in detail [10]. In line with the results of Fearnley et al., the association between PCOS and endometrial cancer was abolished after adjustment for BMI. These examples demonstrate that the association between PCOS and endometrial cancer is well explained by the association of both disorders with obesity [2]. Furthermore, an overall 17-fold higher risk of endometrial cancer was observed in 8155 Taiwanese women with PCOS compared with women without PCOS [11]. The incidence of endometrial cancer was 22.6 and 1.5 per 100,000 person-years in the PCOS and comparison groups, respectively. Once again, risk factors for endometrial cancer, such as BMI, metabolic syndrome, and fertility were not adjusted and were not assessed as potential confounders.

Therefore, data regarding a possible association between PCOS and endometrial cancer should be evaluated after adjustment with other well known risk factors, which are associated with PCOS [12]. The difficulties associated with correctly assessing the relations between PCOS and endometrial cancer are further illustrated by a recent systematic review on this topic [13]. The authors identified 11 individual studies and 3 meta-analyses on the associations between PCOS and endometrial cancer. Multiple studies reported that women with PCOS were at a higher risk for endometrial cancer; however, many did not take into account BMI, a strong and well-established risk factor for endometrial cancer. The interpretation of the data was also limited by the heterogeneity of the studies. For example, some studies evaluated the risk of endometrial cancer in women with polycystic ovaries, androgen excess, and chronic anovulation, whereas others also included menstrual disorders other than PCOS. Some studies included only women < 50 years, who have a very low background rate of cancer, limiting the internal validity of these studies. Furthermore, conditions such as diabetes, metabolic syndrome, and insulin resistance were not always accounted for. The frequent use of OC for the treatment of patients with PCOS was another potential confounder. In light of these methodological difficulties, the authors concluded that the associations between PCOS and endometrial cancer as well as other gynecologic cancers such as ovarian cancer and breast cancer are complex, and there is a need to consider many methodological issues in future analyses. Larger well-designed studies, or pooled analyses, may help clarify these complex associations.

## 4. OC and Risk of Endometrial Cancer

The association between the prolonged use of OC and cancer has been investigated in numerous studies. Although the studies are very heterogeneous, a significant decrease of endometrial cancer incidence has been observed for women using OC compared to never users [14]. In a recent high-quality pooled analysis of 36 epidemiological studies, 27,276 women with endometrial cancer and 115,743 women without endometrial cancer were compared [15]. Overall, 35% of women with endometrial cancer and 39% of controls had ever used OCs. The median duration of OC use was 3 (range 1–7 years) and 4 (range 2–9 years) years for women with endometrial cancer and controls, respectively. The risk of endometrial cancer was significantly lower in ever users than in never users (RR 0.69; 95% CI 0.67–0.72) (Table 2).

The reduction of endometrial cancer risk was greater with a longer use of OCs. Specifically, each 5-year period of use was associated with a risk ratio of 0.76 (95% CI 0.73–0.78) and persisted for more than 30 years. The effect was observed even for OCs with higher estrogen doses used in the 1960s, 1970s, and 1980s. Of note, the effect was only significant for endometrial carcinomas (RR 0.69, 95% CI 0.67–0.72), whereas no risk reduction was observed for sarcomas of the uterus (RR 0.83, 95% CI 0.67–1.04). The protective effect of OC did not vary after adjustment for BMI, parity, menopausal status, smoking, and age at menarche.

The strong and persisting protective effect of OCs regarding endometrial cancer was confirmed in a large retrospective study with a median follow-up of 44 years [16]. In this study, the use of OCs was associated with a 33% reduced incidence of endometrial cancer (RR 0.67; 99% CI 0.66–0.99). The benefit of OCs regarding endometrial cancer persisted for at least 30 years after stopping the intake of OCs.

Michels et al. investigated the influence of various lifestyle characteristics on the protective effect of OCs on endometrial cancer risk. In a cohort study of 2337 women with endometrial cancer and a prior use of OCs, the modulating effect of cigarette smoking, alcohol consumption, BMI, and physical activity was evaluated in detail [17]. In this cohort, the ever use of OCs was associated with a reduced risk of endometrial cancer. Interestingly, however, the use of OCs was only associated with reduced endometrial cancer risk in women with a BMI of 30 or greater (HR 0.36; 95% CI 0.25–0.52), who were current smokers (HR 0.47; 95% CI 0.25–0.88), consumed alcohol (HR, 0.23; 95% CI, 0.07–0.74), and exercised moderately or infrequently (HR 0.50; 95% CI 0.33–0.75). A possible anti-estrogenic interaction of OCs with the specific chemicals in tobacco smoke or in alcohol have been proposed to explain these effects [17]. Since obesity is a strong risk factor for developing endometrial cancer, the risk-reducing use of OCs might be an attractive prevention option for obese women needing contraception [18].

All of the above cited data are supported by a systematic review regarding the use of hormonal contraceptives and the risk of endometrial cancer [14]. This review identified four case–control and five cohort studies evaluating the association between OCs use and endometrial cancer incidence. Of these, three case–control studies with 3,981,072 person-years and four cohort studies with 308,198 women were included in a meta-analysis comparing ever versus never use of OCs. Ever use of OCs protected against endometrial cancer, with an OR of 0.57 (95% CI; 0.43–0.77). However, the heterogeneity between the studies was considerable and should be taken into consideration. Similar results were obtained by an older meta-analysis published by Schlesselman in 1997 [19]. In this meta-analysis of 11 case–control studies, the main conclusion was that use of OCs for at least 4 years was protective, with a longer duration of OC use conferring greater protection (relative risk (RR) 0.44 for 4 years of use; RR 0.33 for 8 years of use; RR 0.28 for 12 years of use). The risk of endometrial cancer in women using a levonorgestrel-releasing intrauterine system as a contraceptive was also investigated. In a population-based prospective cohort analysis, the use of a levonorgestrel-releasing intrauterine system was associated with a reduced risk of endometrial cancer 0.22 (95% CI: 0.13–0.40) in comparison with never use [20]. The most important mechanism by which PCOS increased the risk of endometrial cancer is the prolonged exposure of the endometrium to unopposed estrogen [21], which results from anovulation, commonly observed in patients with PCOS. Hypersecretion of luteinizing hormone and increased expression of its receptors in the endometrial tissue of women with PCOS have also been proposed as risk factors for endometrial cancer [21]. Although the role of insulin resistance is not yet well understood, the resulting hyperinsulinemia has also been associated with increased endometrial cancer risk. These observations suggest again the importance of an elevated BMI as confounder regarding the risk of PCOS for endometrial cancer. A reduced number of ovulations with reduced exposure to naturally occurring female hormones and the protective effect of progestin are common mechanisms associated with the protective effect of OCs.

In conclusion, the risk of endometrial cancer is significantly lower among women who have ever used OCs. This effect persists for at least 30 years after stopping OCs and increases with a longer duration of OC use. In addition to the direct anti-estrogenic effects of synthetic progestogens used in OCs, OC use might also decrease the estrogenic effects of known risk factors for endometrial cancer.

## 5. Tamoxifen and Risk of Endometrial Cancer

TAM is a selective estrogen receptor modulator (SERM) used for the treatment of women with breast cancer. TAM can increase the risk of endometrial pathologies such as endometrial hyperplasia and endometrial carcinoma by its estrogenic activity in this tissue [22]. Specifically, TAM therapy of postmenopausal women with breast cancer is associated with an increased risk of endometrial hyperplasia. In a retrospective study of 238 patients treated with TAM, the risk of simple and complex endometrial hyperplasia was 12% and 3%, respectively [23]. Endometrial cancer was observed in 2% of patients. The rate of atypical endometrial hyperplasia in the large National Surgical Adjuvant Bowel Project (NSABP) P-2 trial, in which TAM was administered for breast cancer prevention, was 0.77 per 1000 women per year [24]. The increased risk of endometrial cancer during the long-term use of TAM was increased by approximately two- to three-fold compared to that in women not administered TAM [23,25]. In a prospective randomized prevention trial, 13,388 women at increased risk for breast cancer were randomly assigned to receive a placebo or TAM for 5 years (Table 3). TAM reduced the risk of invasive breast cancer but increased the risk for endometrial cancer [26].

The relative risk to develop endometrial cancer was 2.53 (95% CI 1.35–4.97) and regarded predominantly in women aged 50 years or older. Notably, all endometrial cancers in the tamoxifen group were limited to the uterus, and no endometrial cancer-related deaths were noted. A follow-up report of this study with a median follow-up of 7 years demonstrated that the risk of endometrial cancer was even higher than previously noted, i.e., about 29% higher than in the original report [27]. After TAM administration, the risk of invasive endometrial cancer was significantly increased (RR 3.28; 95% CI 1.87–6.03). This effect was only observed in patients aged 50 years or older (RR 5.33; 95% CI 2.47–13.17). The cumulative rate of invasive endometrial cancer during the follow-up was 4.68 per 1000 women in the placebo group and 15.64 per 1000 women in the tamoxifen group. In accordance with these results, a meta-analysis of three prospective breast cancer prevention trials calculated the risk of endometrial cancer after TAM administration [28]. The risk of endometrial cancer was elevated in women aged 50 years and older, who received TAM compared to a placebo (RR 1.19; 95% CI 0.53–2.65).

It is well documented that an increased duration of TAM treatment is associated with an increased risk of developing endometrial cancer. For example, in the worldwide Adjuvant Tamoxifen: Longer Against Shorter (ATLAS) trial, 12,894 women with breast cancer who received TAM for 5 years were randomly allocated to continue TAM for another 5 years or to stop treatment (control group) [29]. In this study, the absolute cumulative risk of endometrial cancer was 3.1% and 1.6% in the extended TAM group and in the control group, respectively. Thus, the risk of endometrial cancer after 10 years of TAM was significantly higher compared to the risk of endometrial cancer after 5 years of TAM (RR 1.74; 95% CI 1.30–2.34). A subsequent meta-analysis which pooled the results of ATLAS with another randomized trial also found an increased risk of endometrial cancer after the prolonged use of TAM [22]. The cumulative risk of endometrial cancer increased from 1.5% after 5 years of TAM to 3.2% after 10 years of TAM. The relative risk of endometrial cancer after the extension of TAM was 2.29 (95% CI 1.60–3.28). This effect of TAM was independent of its dosage and became normalized approximately two years after stopping the treatment [30]. An association between the use of TAM and the development of high-risk non-endometrioid endometrial cancer has also been reported [31,32].

The pathophysiological mechanism of TAM-induced endometrial pathology could be explained by the estrogen-agonistic effect of TAM on the endometrium [33], specifically, via the recruitment of various co-regulators in breast tissue leading to the stimulation of cell proliferation and tumor growth. In addition, TAM promotes cytoskeletal remodeling and the migration of endometrial cancer cells. Lastly, DNA damage caused by TAM is an additional mechanism involved in TAM-induced endometrial carcinogenesis. At the molecular level, TAM activates the estrogen receptor (ER) α36 and the downstream G-protein-coupled estrogen receptor-1 (GPER-1) [34]. Our group has recently demonstrated that TAM has a stimulating effect on the membrane-bound estrogen receptor GPER-1 [35]. In a retrospective study of breast cancer patients, we found that the expression of GPER-1 correlated with the time between the diagnosis of endometrial abnormality and the duration of TAM treatment [36].

In contrast to TAM, the selective estrogen receptor modulator raloxifene does not seem to increase the risk of endometrial cancer. In the NSABP STAR/P-2 trial, for example, 4739 women received TAM, and 4717 women received raloxifene [24]. The incidence of endometrial cancer was 45% lower in the raloxifene group compared with the tamoxifen group (RR 0.55; 95% CI 0.36–0.83).

In conclusion, TAM is associated with a significantly increased risk of endometrial cancer, with its longer use increasing the risk. This increased risk of developing endometrial cancer is only evident during the treatment with TAM and no longer after stopping the drug [28].

## 6. Conclusions

In this review, we have summarized the evidence on the association between infertility, PCOS, the use of OCs, and the use of TAM and the risk of endometrial cancer. Although the risk-increasing effect of TAM and the risk-decreasing effect of OCs are evident and well established, the association between infertility and PCOS and endometrial cancer remains controversial, largely due to the unclear interactions with several confounders, and needs to be investigated further.

## Figures and Tables

**Table 1:** Characteristics of the studies included in this review regarding infertility and endometrial cancer risk. aHR, adjusted hazard ratio.

Study	Study Design	Cases	Controls	Risk of Endometrial Cancer	Limitations
Yang et al., 2015	Meta-analysis	8153	11,713	aHR 1.82; 95% CI 1.59–1.94	self-reported infertility; inconsistent infertility definition; infertility treatment is not concerned
Lundberg et al., 2019	Population-based	117,500	2,765,347	aHR 1.25; 95% CI 1.11–1.40	additional hormonal factors and infertility treatment are not concerned
Murugappan et al. 2019	Claims-based cohort	64,345	3,128,345	aHR 1.78; 95% CI 1.39–2.28	Imbalanced distribution of additional risk factors; low rate of infertility; low absolute rate of cancer; short follow-up;

**Table 2:** Characteristics of the studies included in this review regarding the use of oral contraceptives and endometrial cancer risk.

Study	Study Design	Never Users	Ever Users	Risk of Endometrial Cancer	Limitations
Gierrisch et al. 2013	Systematic review	n.a.	n.a.	OR, 0.57; CI, 0.43–0.77	heterogeneity across the studies; variation in the confounding variables between the studies
Colaborative Group on Epidemiological Studies on Endometrial Cancer; 2015	Meta-analysis	87,935	55,084	RR 0.69; 95% CI 0.67–0.72	various duration of use and time since last use of oral contraceptives between studies; confounding due to parity is not concerned
Iversen et al., 2017	Observational	22,920	23,102	RR 0.67; 99%CI 0.66–0.99	additional hormonal factors and hormonal content of the pills as well as parity are not concerned
Michels et al., 2018	Population-based	118,144	78,392	RR 0.66; 95% CI 0.56–0.78	confounding due to parity is not concerned; potential selection bias

**Table 3:** Characteristics of the studies included in this review regarding tamoxifen use and endometrial cancer risk.

Study	Study Design	No Tamoxifen	Tamoxifen	Risk of Endometrial Cancer
Fisher et al., 1998	Prospective randomized	6707	6681	RR 2.53; 95% CI 1.35–4.97
Fisher et al., 2005 (extended follow-up)	Prospective randomized	6610	6597	RR 3.28; 95% CI 1.87–6.03
		5 years Tamoxifen	10 years Tamoxifen	
Davies et al., 2013	Prospective randomized	3418	3428	RR 1.74; 95% CI 1.30–2.34
Fleming et al., 2018	Meta-analysis	7642	7652	RR 2.29; 95% CI 1.60–3.28
